# The crescent sign—a predictor of hip instability in magnetic resonance arthrography

**DOI:** 10.1093/jhps/hnab067

**Published:** 2021-08-21

**Authors:** Corinne A Zurmühle, Valerie Kuner, James McInnes, Dominik H Pfluger, Martin Beck

**Affiliations:** Department of Orthopaedic Surgery and Traumatology, Luzerner Kantonsspital, Luzern, Switzerland; Department of Orthopaedic Surgery and Traumatology, HFR Cantonal Hospital, University of Fribourg, Fribourg, Switzerland; Department of Orthopaedic Surgery and Traumatology, Luzerner Kantonsspital, Luzern, Switzerland; Department of Orthopaedic Surgery, ReBalanceMD, Victoria, British Columbia, Canada; Numerics Data, Ramersberg, Switzerland; Department of Orthopaedic Surgery and Traumatology, Luzerner Kantonsspital, Luzern, Switzerland; Orthopaedic Clinic Lucerne, Hirslanden St. Anna Hospital, Luzern, Switzerland

## Abstract

Currently, much is debated on the optimal treatment of borderline hips, being in the continuum between stable and unstable hips. The diagnosis of stability is often difficult but is a prerequisite for further treatment. Analysis includes a variety of radiographic parameters. We observed that unstable hips often had a crescent-like gadolinium collection in the postero-inferior joint space. We therefore questioned if the ‘crescent sign’ could be an indicator for hip instability? A retrospective comparative study was conducted including 56 hips in the instability group (treated with PAO) and 70 hips with femoroacetabular impingement (FAI) as control group. Based on standard radiographic parameters and magnetic resonance imaging (MRI), the association between hip instability and the ‘crescent sign’ was analyzed. For univariate group comparisons, the non-parametric Wilcoxon two sample test was used. Association between discrete variables was examined by means of chi-square tests. To examine predictive variables, logistic regression models were carried out. Most hips with a crescent sign belong to the instability group. A crescent sign has a sensitivity of 73.3% and specificity of 93% for instability. Based on our results, the crescent sign is a factor that is more prevalent in unstable hips. However, its absence does not exclude instability of the hip. If present, the specificity speaks strongly in favor for instability of the hip.

## INTRODUCTION

The natural history of hip dysplasia is well documented [1, 2] and is associated with the early onset of osteoarthritis. Reduced coverage results in excessive loading of the acetabular rim leading to joint degeneration [3]. Surgical correction of this unfavorable morphology can be successfully achieved by reorienting the acetabulum, most commonly through the use of the Bernese periacetabular osteotomy (PAO) [4]. Improved coverage of the femoral head reduces shear stress and leads to a more physiologic distribution of the joint reaction forces. Long term survivorship and good clinical results support the use of the PAO in hip dysplasia [5]. While the indications for acetabular reorientation in hips with clear radiographic signs of dysplasia with a reduced lateral center edge angle (LCEA) < 20° [6] and acetabular index (AI) of > 10–14° [7–9] are well established, decision-making becomes much more difficult in hips with borderline acetabular coverage radiologically defined with an LCE of 20–25° [6, 10]. The acetabular coverage is only slightly reduced, and it may be difficult to determine the stability of the hip. Unstable, symptomatic borderline dysplastic hips should be treated with a PAO, whereas stable hips are treated according to their underlying pathology.

Classifying a borderline hip as stable or unstable can be challenging [11]. Several radiographic parameters are associated with instability including a low LCEA, a high AI, a high neck shaft angle, increased femoral torsion (FT), upsloping lateral sourcil [12] or a positive Femoro-Epiphyseal Acetabular Roof (FEAR) index [13]. A break in Shenton’s line is indicative of hip subluxation and instability [14]. In magnetic resonance arthrography (MRA) hip instability can be associated with chondral and labral disorders. Hypertrophy of the labrum [15–17] or femoroacetabular hypertrophy of the cartilage has been identified as a sign of hip dysplasia. Further an increased size of iliocapsularis muscle [18, 19], a fovea alta that represents an abnormal superior position of the fovea capitis [20] or tears of the ligamentum teres [21] can be visualized in MR imaging as indirect signs of instabiliy of the hip joint. Some other signs are visible during arthroscopy and give further intraoperative information as for hip dysplasia like the ‘inside-out’ chondrolabral flap or chondrolabral lesions Outerbridge Grade III or IV in the weightbearing area in borderline dysplastic hips [22].

During clinical practice, we observed that unstable hips often had a gadolinium collection in the postero-inferior joint space (‘crescent sign’). Based on that observation we questioned: (i) if the so-called crescent sign is a predictor for hip instability and (ii) if its predictive power can be increased in combination with other established radiographic parameters to identify hip instability?

## MATERIAL AND METHODS

**Fig. 1.**
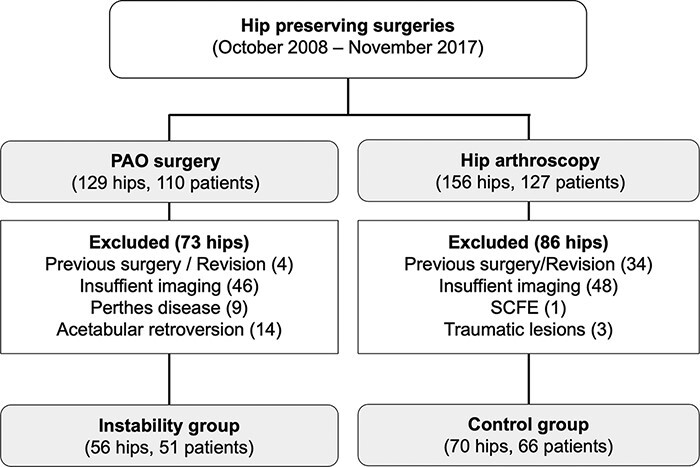
Flowchart of selected patients.

This study was approved by the local ethics committee (Ethikkommission Nordwest- und Zentralschweiz, 2020-00587). A retrospective, comparative study was conducted using the database of hip preserving surgeries performed at our institution between January 2010 and December 2017 (881 hips). All patients were treated by a single orthopedic surgeon (M.B.). One hundred and ten patients (129 hips) had a PAO and 442 patients (482 hips) underwent arthroscopic FAI correction. Of these, all hips who had a PAO for symptomatic hip instability with an LCE angle < 20° and AI > 10° or unstable symptomatic borderline dysplasia with an LCE between 20 and 25° were located into the instability group. In our daily practice, the FEAR-index and presence of a hypertrophic labrum were used as additional radiological factors. Typical clinical signs of overload of abductor muscles and a positive apprehension sign were used for the decision-making process. The control group consisted of stable hips treated for FAI. The number of hips in the control group was adjusted to the number of hips in the instability group. Cases were reviewed chronologically from the most recent to the most distant until an equal number of cases was reached. 110 patients (129 hip) with a PAO and 127 patients (156 hips) with FAI represented the base of the study. Exclusion criteria for both groups included previous surgery of the hip (instability group 4 hips, control group 34 hips), inadequate imaging e.g. missing radial sequence, insufficient image quality (instability group 46 hips, control group 48 hips), slipped capital femoral epiphysis (control group 1 hip), Legg-Calvé-Perthes disease (instability group 9 hips), treatment of traumatic lesions (control group 3 hips) and acetabular retroversion (instability group 14 hips). The remaining cases for analysis consisted of 56 hips (51 patients) in the instability group and 70 hips (66 patients) in the control group (Fig. 1).

In both study groups, we evaluated preoperative radiographs and MRA assessing following parameters [15]: Joint degeneration, using Tönnis’ classification [23] and FEAR-index [13], was assessed on standardized anteroposterior (AP) pelvic radiographs. LCEA, AI, extrusion index (EI), retroversion index, anterior coverage, posterior coverage and total coverage were measured on the preoperative standardized AP pelvic radiographs using the validated software Hip^2^Norm (University of Bern, Bern, Switzerland) [24–26]. Based on the MR imaging, FT was measured according to the method of Murphy *et al*. [27] and the alpha angle to detect the femoral neck offset according to the method of Nötzli *et al*. [28].

MRA followed a standardized protocol as described previously [15]. Briefly, the protocol for MRA included a transverse T1-weighted turbo spin-echo sequence, a coronal and sagittal intermediate-weighted turbo spin-echo sequence, a radial intermediate-weighted turbo spin-echo sequence, a transverse THRIVE (T1-weighted high-resolution isotropic volume examination) or DESS (dual-echo steady state) sequence and a rapid transverse T1-weighted sequence over the proximal and distal femur for anteversion measurement.

**Fig. 2.**
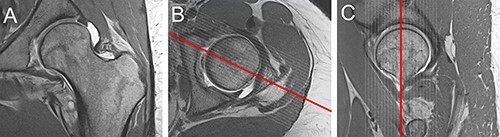
Femoral head center is detected in the three planes: (A) coronal, (B) axial, (C) sagittal. Based on that, the further analysis is performed.

**Fig. 3.**
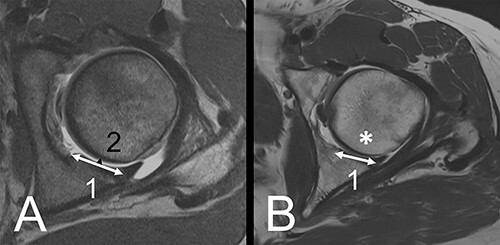
A/B: Axial plane. (A) Example of a dysplastic hip. Posterior horn (1) at the level of the femoral head center. Gadolinium in between the posterior horn and femoral head (2), representing a positive crescent sign. (B) Control hip without a crescent sign.

**Fig. 4.**
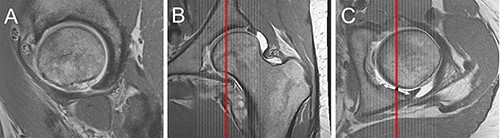
A–C: Sagittal plane at the level of the center of the posterior wall (CPW). The crescent sign was assessed on the sagittal plane (A) going through the CFH and the center of posterior wall (C).

**Fig. 5.**
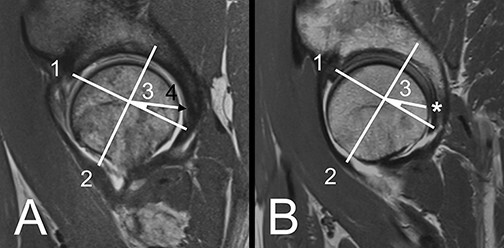
A/B: Sagittal plane measurement at CFH. (A) Instable, borderline dysplastic hip. A line is drawn between the edge of the anterior and posterior wall (1). At mid-distance a perpendicular line is drawn (2). From the intersection an angle of 22.5° (postero-inferior quarter) in the direction to the posterior wall is made (3). (B) Sagittal plane at CFH in a control hip.

**Fig. 6.**
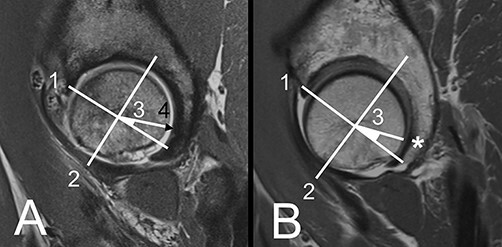
A/B: (A) Instable, borderline dysplastic hip. Crescent sign on the sagittal slice through CPW. Identification of location to assess the presence of a crescent sign is performed like in Fig. 5 but at the level of CPW. Number (4) shows the crescent sign in this plane in with a black triangle. (B) Absence of crescent sign in a control hip.

**Fig. 7.**
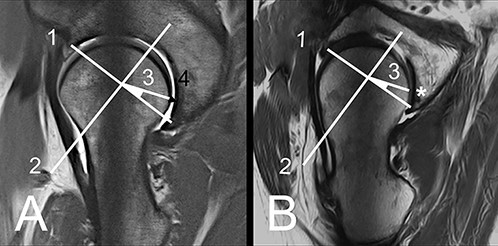
A/B: Radial plane. (A) Instable, borderline dysplastic hip. A line between anterior and posterior wall is defined (1). Then a perpendicular (2) is drawn at the mid-distance is placed. From the intersection a line is made at an angle of 22.5° towards the posterior horn (3). Positive crescent sign (4). (B) Radial plane without a crescent sign in a control hip.

The measurement of the crescent sign was carried out in a standardized way by two observers (J.M. and V.K.) independently. Following four imaging planes were used: axial, two different sagittal and radial. The imaging planes were linked and the slices at the level of the center of femoral head (CFH) were used in all planes for measurement. A crescent sign was assessed as present whenever contrast agent could be seen between femoral head and acetabulum. The planes were defined such that they all are going through the center of the posterior horn. The technique was as follows:

- The four planes were linked and the slice through the CFH identified on each. The resulting axial and sagittal images were used for the first three measurements (Fig. 2a–c).
- Axial plane: The center of the posterior wall (CPW) is identified as the midpoint between the edge of the fossa and the rim of the posterior wall (Fig. 3a and b).
- Sagittal plane: Two measurements were done here—the first at the center of the femoral head CFH. The second at the CPW, to account for possible lateral migration of the femoral head (Fig. 4a–c). A line is drawn from the anterior to the posterior acetabular rim. Then, a perpendicular line is drawn at mid-distance to divide the femoral head. From the intersection a line is drawn at 22.5°, reaching the center of the posterior horn (Fig. 5a and b, Fig. 6a and b), where we see the ‘crescent sign’ mostly.
- Radial plane: With the three other linked images on the CFH, the radial slice which intersects the CPW is then selected (Fig. 7a and b).

Inter-observer agreement was analyzed by adopting the method according to Shrout and Fleiss using the intra-class correlation coefficient (ICC) as a principle measure of reliability [29, 30]. Agreement of the intra-class correlation values was interpreted as: greater than 0.75 = excellent, 0.40–0.75 = fair to good and less than 0.40 = poor [29]. The inter-rater agreement is demonstrated by a graphical technique suggested by Bland and Altman [31].

Univariate group comparisons were carried out using the non-parametric Wilcoxon Two Sample test. Test on normality was carried out by means of the Shapiro–Wilk test. Association between discrete variables was examined by means of chi-square tests. To examine predictive variables with respect to group classification (unstable vs. control), logistic regression models were carried based on a stepwise variable selection.

For all statistical analyses the SAS software, version 9.4 was used (SAS Institute, Cary, NC).

## RESULTS

There were no significant differences according to age, gender or affected side between the two study groups (Table I). Joint degeneration of a maximum of Tönnis grade I was visible without a significant difference between the groups.

**Table I.** Demographic data

*Parameter*	*Instability group*	*Control group*	*P* *-value*
Number of patients	51	66	–
Number of hips	56	70	–
Age (years)	29 ± 10 (15–63)	32 ± 12 (14–54)	0.141
Gender (% male of all hips)	71	56	0.070
Side (% right of all hips)	64	49	0.056

Values are expressed as mean ± standard deviation with range in parentheses.

The ICC for all analyzed parameters demonstrated excellent agreement (ICC 0.85–0.99, excellent >0.75), with the highest level for the crescent sign demonstrated in the axial plane with an ICC of 0.99.

There was no significant difference in degree of arthritis between groups using the Tönnis score. As expected, the instability group was found to have reduced LCEA and acetabular coverage, elevated EI, AI and a pathological FEAR-index > 2 compared to the control group with stable hips. With exception of the FT and the retroversion index, which were equal and normal in the two study groups, all parameters between the two groups showed a significant difference (Table II). Both groups had 13 hips with borderline cover (LCEA ≥ 20 and <25°), one hip in the control group had an LCEA of 19°.

**Table II.** Hip parameters of the two study groups

*Parameter*	*Normal value*	*Instability group*	*Control group*	*P* *-value*
x-ray-based measurements				
LCEA (degrees) [6]	25–33	16 ± 7 (3–29)	31 ± 6 (19–47)	<0.0001
AI (degrees) [35]	0–10	19 ± 7 (4–39)	6 ± 4 (−5–17)	<0.0001
EI (degrees) [7]	17–27	33 ± 7 (18–48)	20 ± 6 (6–34)	<0.0001
Retroversion index (percent) [36, 37]	<4	8 ± 15 (0–47)	12 ± 15 (0–51)	0.170
Anterior coverage (percent) [7]	15–26	13 ± 7 (0–33)	24 ± 9 (6–61)	<0.0001
Posterior coverage (percent) [7]	36–47	34 ± 11 (7–57)	44 ± 9 (27–72)	<0.0001
Total coverage (percent) [7]	70–83	63 ± 9 (43–79)	80 ± 7 (63–93)	<0.0001
FEAR-index (degrees) [13]	<2	4 ± 11 (−28–27)	−16 ± 8 (−41–6)	<0.0001
Tönnis score (percent) [23]				0.414
Grade 0		89 (50 hips)	87 (61 hips)	
Grade 1		11 (6 hips)	13(9 hips)	
Grade 2		0	0	
Grade 3		0	0	
MR-based measurements				
Alpha angle (degrees) [28]	<50	50 ± 12 (31–90)	63 ± 12 (34–84)	<0.0001
FT (degrees) [23, 27]	10–25	24 ± 12 (−4–50)	20 ± 9 (−1–42)	0.052

Values are expressed as mean ± standard deviation with range in parentheses.

The majority of patients with a positive crescent sign belong to the group with unstable hips. In 37 hips (29.4%, 33 hips instability group, 4 hips control group), the sign was present. When a crescent sign is present in all planes, an acceptable sensitivity of 73.3% and good specificity with 93% for the presence of instability was found (Table III). Of all measurements of the newly introduced crescent sign (radial, sagittal CFH/CPW, axial), the most sensitive individual measurement was the axial [67.9%, negative predictive value 77% (CI 95% 67 to 85)] (Table IV), whereas the most specific was the sagittal at the CFH [94.3%, positive predictive value 84% (CI 95% 64 to 95)] (Table V). The strongest association of an instability was seen when the crescent sign was present in all slices (Table VI).

**Table III.** Summary of statistical measures of FEAR-index and crescent signs parameters

		*Crescent signs*				
*Parameter*	*FEAR-Index*	*Axial*	*Radial*	*Sagittal (CFH)*	*Sagittal (CPW)*	*Signs present in all planes*
Sensitivity (%)	65.5	67.9	66.1	39.3	66.1	73.3
Specificity (%)	95.7	88.6	84.3	94.3	87.1	93

CEFH = Center of Femoral Head; CPW = Center of Posterior Wall.

**Table IV.** Diagnostic performance of crescent sign in axial plane with the highest predictive power

*Parameter*	*Instability group* *—* *unstable hips (n)*	*Control group* *—* *stable hip (n)*	
Crescent sign absent	18	62	Negative predictive value: 77% (CI 95% 67 to 86)
Crescent sign present	38	8	Positive predictive value: 83% (CI 95% 68 to 92)
	Sensitivity: 68% (CI 95% 54 to 80) False negative rate: 22% (CI 95% 14 to 33)	Specificity: 89% (CI 95% 78 to 95) False positive rate: 17% (CI 95% 8 to 32)	

CI = confidence interval.

**Table V.** Diagnostic performance of crescent sign in sagittal plane with the highest predictive power

*Parameter*	*Instability group* *—* *unstable hips (n)*	*Control group* *—* *stable hip (n)*	
Crescent sign absent	4	66	Negative predictive value: 66% (CI 95% 56 to 75)
Crescent sign present	22	34	Positive predictive value: 84% (CI 95% 64 to 95)
	Sensitivity: 39% (CI 95% 27 to 53) False negative rate: 34% (CI 95% 25 to 44)	Specificity: 94% (CI 95% 85 to 98) False positive rate: 15% (CI 95% 5 to 36)	

CI = confidence interval.

**Table VI.** Association between groups and ‘crescent sign’

*Crescent sign (axial, radial, sagittal)*	*Instability group*	*Control group*	*Total*
No, No, No	12 (18.5%)	53 (81.5%)	65 (51.6%)
No, No, Yes	4 (57.1%)	3 (42.9%)	7 (5.6%)
No, Yes, No	2 (33.3%)	4 (66.7%)	6 (4.8%)
No, Yes, Yes	0 (0.0%)	2 (100.0%)	2 (1.6%)
Yes, No, No	1 (50.0%)	1 (50.0%)	2 (1.6%)
Yes, No, Yes	2 (50.0%)	2 (50.0%)	4 (3.2%)
Yes, Yes, No	2 (66.7%)	1 (33.3%)	3 (2.4%)
Yes, Yes, Yes	33 (89.2%)	4 (10.8%)	37 (29.4%)
Total	56 (44.4%)	70 (55.6%)	126 (100.0%)

Chi-square statistic: 50.81, df = 7, *P*-value < 0.0001. Note: %fractions in the inner cells are calculated by row.

In 51.6% of all hips, the crescent sign was absent on all three planes, including 12 hips (18.5%; 6 dysplastic, 6 borderline hips) that were classified as unstable and the rest being stable hips. Conversely, if all three crescent signs were present 89.2% of the hips were from the instability group. This was the case in 29.4% hips. The remaining inconsistent hips make out 19% [(100 − (51.6 + 29.4) = 19%] with an incidence of instability ranging from 0 to 66.7%. In this small subgroup, it is more difficult to judge the risk of instability. However, it would seem that a present crescent sign on the sagittal CFH measurement may predict a higher likelihood of instability, while its absence on axial imaging indicates a lower risk. In the control group, 12 hips had a crescent sign on at least one plane. Nine were well covered hips (LCEA 28 to 40°), three hips had an LCEA of 23, 24 and 25°.

An evaluation of the sub-population of borderline hips with chi-square-test with an LCE of 20–25° revealed the presence of a crescent sign in the axial plane in 16.7% of hips in FAI group (2 of 12 hips) and 57% in PAO group (8 of 14 hips) with a *P*-value of 0.0511. Analysis of sagittal plane in MPW showed 0.8% of hips in FAI group (1 of 12 hips) and 50% in PAO group (7 of 14 hips) positive for the crescent sign with a significant *P*-value of 0.0357. In the sagittal plane CEFH (FAI group 0% with 0 of 12 hips, PAO group 29% with 4 of 14 hips, *P*-value 0.100) and the radial plane (FAI group 42% with 5 of 12 hips, PAO group 36% with 5 of 14 hips, *P*-value 1.000), there was no significant difference seen.

However, regarding the sub-population of borderline hips, in 33.3% of the FAI cases a crescent sign was present in at least one plane, but in 57.1% in PAO cases (*P*-value 0.659).

A multivariate analysis was performed addressing the question whether a combination of the crescent sign with other radiographic parameters [LCEA, EI, AI and FEAR-index, as well as the acetabular coverage (total, anterior, posterior)] can improve the prediction of instability. Using a stepwise regression model, the variables LCEA, EI and AI turned out to be significant and predictive (*P* < 0.05).

The optimal separation of probability levels was about at *P* = 0.400 with highest sensitivity and specificity. For 1° increase of the AI, the risk of instability increases by factor 1.57 whereas for LCE with 1° increase the risk reduces by factor 0.65. Modeling based on categorized predictors and the results of partition analysis revealed cut-off values of 11° for AI and 23° for LCEA, respectively. For cases with AI above 11°, the risk of instability increases by factor 76, whereas for cases having LCEA values above 23° the risk reduces by factor 0.07.

## DISCUSSION

Hip dysplasia represents a spectrum of disease from frank dislocation of the femoral head from an underdeveloped acetabulum to mild borderline dysplasia that may only be detected through detailed examination of multiple radiographic parameters. Symptomatic patients may have features that could be consistent with borderline dysplasia or FAI which complicates treatment decisions. Identifying which features predominate may dictate the most appropriate treatment and avoid insufficient outcome and complications. For example, a hip with borderline dysplasia that is treated for FAI and labral reattachment could result in iatrogenic worsening of instability, leading to progression of symptoms and joint degeneration. Conversely, an impinging hip treated with an acetabular reorientation procedure could result in worsened impingement, if the cam deformity is not corrected at time of surgery, as proposed by Albers *et al*. [32]. As such, any additional cues to help detect instability may improve the ability to choose the most appropriate treatment strategies in these subtle cases. We submit that the crescent sign on MRA may be an indicator of instability in borderline hips and may be a useful adjunct in conjunction with other accepted parameters. Therefore, MRA can add important information for hip instability, which would be missed on a standard MRI.

**Fig. 8.**
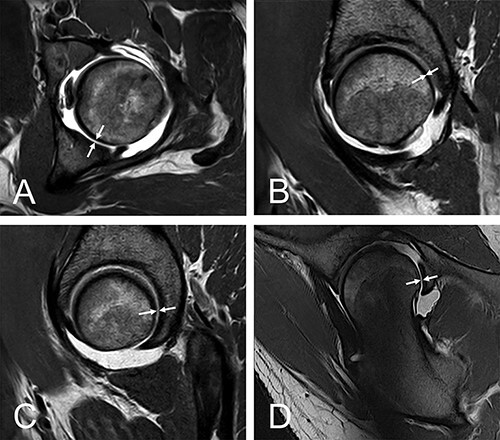
Example of a 28-year-old female with borderline hip dysplasia with positive crescent signs in axial (A), sagittal CPW (C), radial and (D) planes. Negative crescent sign in sagittal plane CFH (B).

Our study results indicate that the crescent sign is a factor that seems to be more prevalent in unstable hips. If present, particularly in the sagittal plane at the center of the femoral head, the crescent sign strongly suggests the presence of hip instability based on its high specificity. Its absence, however, given the weaker sensitivity, does not exclude instability of the hip. The relatively high specificity in both the axial and sagittal CFH planes are consistent with the typical direction of migration of the hip in dysplasia being ‘up and out’. Overall, it could be shown that the highest sensitivity could be seen if the crescent sign was detected in all three planes. The reduction of the measurements to one plane only would result in reduced statistical power and sensitivity, why we recommend the assessment in all planes. We assume that the pooling of gadolinium in the posterior aspect of the hip joint is due to the phenomenon that unstable hips migrate in a lateral and anterior direction, giving space posteriorly. This migration cannot be seen radiologically, unless in major instability where a break in Shenton’s line can be observed [14]. This concept is supported by the work of Henak *et al.* [33] who showed with finite element models that the loaded dysplastic hip achieved equilibrium near the lateral edge of the acetabulum. Another possibility could be a curvature mismatch between femoral head and acetabulum. This recently was described as possible cause for hip instability [34]. This was shown to be increased in borderline and dysplastic hips with the acetabular radius being larger allowing some play giving room for gadolinium between the joint surfaces as was observed in this study (Fig. 8).

Although we attribute the presence of a crescent sign with instability of the hip, there were 12 hips (19%; 6 dysplastic, 6 borderline hips) who underwent a PAO that did not show a crescent sign on any of the four planes. On the other hand, it was present in the control group in twelve hips of which nine were well covered (LCEA 28 to 40°). Whether in the well-covered hips the presence of a crescent sing is attributed to capsular laxity can only be speculated.

The prevalence of the crescent sign in the sub-population with borderline hips (12 in FAI group and 14 in PAO group) shows a tendency of being more presence especially in sagittal MPW and axial plane and could help to detect an instability of the hip. However, it is difficult to generalize because of the small numbers of patients in both groups.

The absence of a crescent sign in unstable hips may demonstrate that soft tissue constraints may be adequate in some cases to prevent migration in the supine position. As migration of the hip is a dynamic process, one could expect that subluxation may best be viewed in a standing position with the limb loaded. This may demask some of the unstable hips without crescent signs. Future comparisons with MR-A studies obtained in a standing MRI could be valuable to investigate this possibility.

Due to its relatively limited sensitivity (39.3–67.9% in individual planes, 73.3% if present in all planes), we must caution that the absence of a crescent sign should be used to rule out instability. Its high specificity in both the sagittal CFH (94.3%) and axial (88.6%), as well as if present in all planes (93%), however, supports its use as an additional factor that should be observed and included in the decision-making process in the treatment of borderline hips with subtle instability.

**Fig. 9.**
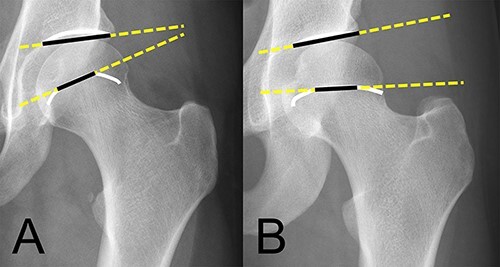
The FEAR-index is positive in case where the angle between the acetabular roof hand the line through the most medial and most lateral point of the central part of the epiphysis shows a lateral directed angle. Compared to a negative FEAR-Index, where the angle is medially directed with the apex formed by the femoral epiphysis and the AI pointing laterally [13]. (A) shows a negative FEAR-index and (B) shows a borderline dysplastic hip with a positive FEAR-index.

**Fig. 10.**
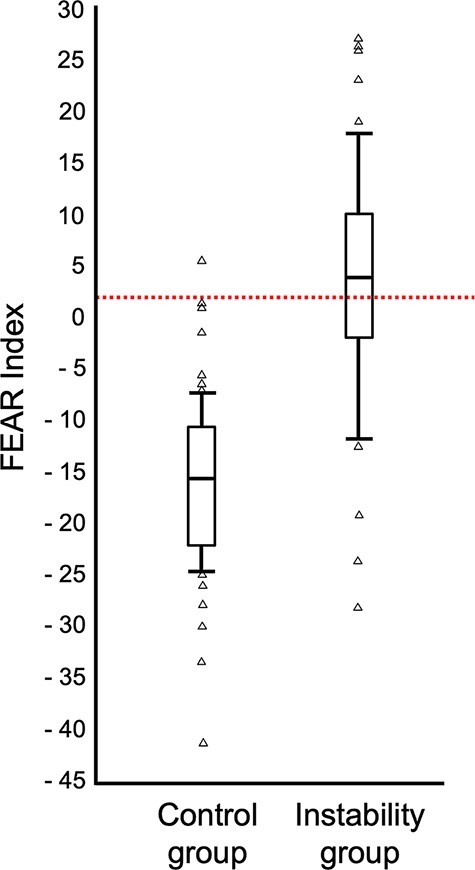
Boxplot FEAR-Index for control and instability group.

Comparing these results with the FEAR-index (sensitivity of 65.5%), the crescent sign shows the same tendency, with an excellent specificity but relatively limited sensitivity. Wyatt *et al*. [13] suggested that the FEAR-index appeared to have a role in clarifying borderline hips that would behave as stable. The crescent sign, in comparison, seems better suited based on our study to identify hips that behave as unstable. The combined assessment of the FEAR-index on plain radiography and the presence or absence of the crescent sign of MRA may guide surgical treatment by helping to clarify the predominant pathology of borderline hips as over constrained or unstable. Our paper further supported the validity of the FEAR-index (Fig. 9) with similar measurements reported in a far greater number of patients than initially studied (Fig. 10).

Our study has several limitations. The presence of a crescent sign may be influenced by several factors like the injected volume of gadolinium contrast agent, joint laxity, missing labral seal, cartilage wear or the positioning of the legs during examination. Before assessing crescent sign, radiographic joint space narrowing, antero-superior cartilage wear of the acetabulum and femoral head has to be excluded. In those situations, the femoral head may migrate antero-superiorly, leading to a crescent sign. Similarly, a contre-coup lesion, as seen in pincer FAI, may lead to an accumulation of gadolinium in the posterior joint area in the postero-inferior area of cartilage loss. Furthermore, the femoral head may occasionally be flattened posteriorly, and this asymmetry relative to the acetabulum may provide space for pooling of contrast dye.

In conclusion, the presented study introduces the crescent sign as an additional radiographic parameter to clarify decision-making in borderline hips. In symptomatic patients with these types of hips, a positive crescent sign supports the possibility of instability as the predominant pathology. Based on its high specificity when present, a crescent sign supports a stability focused treatment such as a PAO to improve coverage. It should not be considered conclusive on its own but coupled with additional parameters may help focus treatment decisions.

## Data Availability

Data available on request.

## LEVEL OF EVIDENCE

Level III, diagnostic study.
